# Retraction: Equilibrium and kinetic studies on adsorptive removal of malachite green by the citrate-stabilized magnetite nanoparticles

**DOI:** 10.1039/d0ra90084b

**Published:** 2020-08-13

**Authors:** Laura Fisher

**Affiliations:** Royal Society of Chemistry Thomas Graham House, Science Park, Milton Road Cambridge CB4 0WF UK advances-rsc@rsc.org

## Abstract

Retraction of ‘Equilibrium and kinetic studies on adsorptive removal of malachite green by the citrate-stabilized magnetite nanoparticles’ by Rashmi Rani Mishra *et al.*, *RSC Adv.*, 2014, **4**, 51787–51793, DOI: 10.1039/C4RA07651F.

The Royal Society of Chemistry hereby wholly retracts this *RSC Advances* article due to concerns with the reliability of the data in the published article.

Repeating fragments can be observed in the XRD spectrum in Fig. 1D, which indicates that it has been manipulated. In addition, there are a number of identical particles replicated within the TEM image that was used in the graphical abstract suggesting that the image has also been manipulated. Given the significance of the concerns about the validity of the data, the findings presented in this paper are no longer reliable.

S. Sudheer Khan opposes this retraction. Rashmi Rani Mishra and Preethy Chandran were contacted but did not respond.

Signed: Laura Fisher, Executive Editor, *RSC Advances*

Date: 28^th^ July 2020

## Supplementary Material

